# Predicting the outcome of COVID-19 infection in kidney transplant recipients

**DOI:** 10.1186/s12882-021-02299-w

**Published:** 2021-03-19

**Authors:** Ozgur Akin Oto, Savas Ozturk, Kenan Turgutalp, Mustafa Arici, Nadir Alpay, Ozgur Merhametsiz, Savas Sipahi, Melike Betul Ogutmen, Berna Yelken, Mehmet Riza Altiparmak, Numan Gorgulu, Erhan Tatar, Oktay Ozkan, Yavuz Ayar, Zeki Aydin, Hamad Dheir, Abdullah Ozkok, Seda Safak, Mehmet Emin Demir, Ali Riza Odabas, Bulent Tokgoz, Halil Zeki Tonbul, Siren Sezer, Kenan Ates, Alaattin Yildiz

**Affiliations:** 1 Division of Nephrology, Department of Internal Medicine, Istanbul University Istanbul Faculty of Medicine, Istanbul, Turkey; 2 Department of Nephrology, University of Health Sciences, Haseki Training and Research Hospital, Istanbul, Turkey; 3 Division of Nephrology, Department of Internal Medicine, Mersin University Faculty of Medicine, Training and Research Hospital, Mersin, Turkey; 4 Department of Nephrology, Hacettepe University Faculty of Medicine, Ankara, Turkey; 5 Division of Nephrology, Memorial Hizmet Hospital Department of Internal Medicine, İstanbul, Turkey; 6 Division of Nephrology, Department of Internal Medicine, Yeni Yuzyil University Faculty of Medicine, Istanbul, Turkey; 7 Division of Nephrology, Department of Internal Medicine, Sakarya University Faculty of Medicine, Training and Research Hospital, Sakarya, Turkey; 8 Department of Nephrology, University of Health Sciences, Haydarpasa Numune Training and Research Hospital, Istanbul, Turkey; 9 Department of Transplantation, Koc University Hospital, Istanbul, Turkey; 10 Division of Nephrology, Department of Internal Medicine, Istanbul University - Cerrahpasa Cerrahpasa Faculty of Medicine, Istanbul, Turkey; 11 Division of Nephrology, Department of Internal Medicine, University of Health Sciences, Bagcilar Training and Research Hospital, Istanbul, Turkey; 12 Department of Nephrology, University of Health Sciences, Izmir Bozyaka Training and Research Hospital, Izmir, Turkey; 13 Department of Nephrology, Bursa City Hospital, Bursa, Turkey; 14 Department of Nephrology, Darica Farabi Training and Research Hospital, Kocaeli, Turkey; 15 Division of Nephrology, Department of Internal Medicine, Sakarya University Training and Research Hospital, Sakarya, Turkey; 16 Şişli Memorial Hospital Department of Internal Medicine, Division of Nephrology, İstanbul, Turkey; 17 Department of Nephrology, University of Health Sciences, Sultan 2, Abdulhamid Han Training and Research Hospital, Istanbul, Turkey; 18 Department of Nephrology, Erciyes University Faculty of Medicine, Kayseri, Turkey; 19 Department of Nephrology, Necmettin Erbakan University Meram Faculty of Medicine, Konya, Turkey; 20 Division of Nephrology, Department of Internal Medicine, Atilim University Faculty of Medicine, Istanbul, Turkey; 21 Division of Nephrology, Department of Internal Medicine, Ankara University Faculty of Medicine, Ankara, Turkey

**Keywords:** Kidney transplantation, COVID-19, Registry

## Abstract

**Background:**

We aimed to present the demographic characteristics, clinical presentation, and outcomes of our multicenter cohort of adult KTx recipients with COVID-19.

**Methods:**

We conducted a multicenter, retrospective study using data of patients hospitalized for COVID-19 collected from 34 centers in Turkey. Demographic characteristics, clinical findings, laboratory parameters (hemogram, CRP, AST, ALT, LDH, and ferritin) at admission and follow-up, and treatment strategies were reviewed. Predictors of poor clinical outcomes were analyzed. The primary outcomes were in-hospital mortality and the need for ICU admission. The secondary outcome was composite in-hospital mortality and/or ICU admission.

**Results:**

One hundred nine patients (male/female: 63/46, mean age: 48.4 ± 12.4 years) were included in the study. Acute kidney injury (AKI) developed in 46 (42.2%) patients, and 4 (3.7%) of the patients required renal replacement therapy (RRT). A total of 22 (20.2%) patients were admitted in the ICU, and 19 (17.4%) patients required invasive mechanical ventilation. 14 (12.8%) of the patients died. Patients who were admitted in the ICU were significantly older (age over 60 years) (38.1% vs 14.9%, *p* = 0.016). 23 (21.1%) patients reached to composite outcome and these patients were significantly older (age over 60 years) (39.1% vs. 13.9%; *p* = 0.004), and had lower serum albumin (3.4 g/dl [2.9–3.8] vs. 3.8 g/dl [3.5–4.1], *p* = 0.002), higher serum ferritin (679 μg/L [184–2260] vs. 331 μg/L [128–839], *p* = 0.048), and lower lymphocyte counts (700/μl [460–950] vs. 860 /μl [545–1385], *p* = 0.018). Multivariable analysis identified presence of ischemic heart disease and initial serum creatinine levels as independent risk factors for mortality, whereas age over 60 years and initial serum creatinine levels were independently associated with ICU admission. On analysis for predicting secondary outcome, age above 60 and initial lymphocyte count were found to be independent variables in multivariable analysis.

**Conclusion:**

Over the age of 60, ischemic heart disease, lymphopenia, poor graft function were independent risk factors for severe COVID-19 in this patient group. Whereas presence of ischemic heart disease and poor graft function were independently associated with mortality.

## Introduction

The novel coronavirus 2019 disease (COVID-19), which originated in the city of Wuhan, in Hubei province, China, infected more than 33 million people and caused nearly 1 million reported deaths worldwide (https://coronavirus.jhu.edu/map.html). Studies addressing the risk factors, clinical features, and prognosis of the disease have been published [1, 2]. Approximately 20% of COVID-19 patients have been reported to have moderate to severe clinical manifestations and 5% progress to critical illness [3]. The case fatality rates vary in different reports. In general, it ranged from 1 to 7.2% and reached 49% among patients with critical illnesses [3, 4]. The presence of comorbidities such as old age and diabetes mellitus, hypertension, chronic kidney disease, morbid obesity, coronary heart disease, and chronic lung disease have been identified as major risk factors for severe disease [5]. However, the diagnosis and clinical course of the disease in solid organ transplant (SOT) recipients may differ from the general population due to chronic immunosuppression and coexisting conditions [6]. There is scarce information on the infectious course of COVID-19 in transplant recipients. Although there are currently a couple of reports of COVID-19 among kidney transplant (KTx) recipients [7–9], it is yet unclear whether the presence of immunosuppression increases the complications of COVID-19 [10]. Previous reports suggest that immunosuppression may reduce the frequency of cytokine storms, a significant cause of mortality [11, 12]. We aimed to present the clinical manifestations, course of the disease and outcomes of a large multicenter cohort of adult KTx recipients with COVID-19 in this study. We also examined the predictors of worse clinical outcomes in this group of patients.

## Methods

### Study design and participants

This multicenter, retrospective cohort study was conducted using data collected from 34 centers in Turkey under the unconditional support of the Turkish Society of Nephrology. One hundred nine patients (63 males, 46 females, mean age 48.4 ± 12.4 years old) were included between April 17 and June 1, 2020. The diagnosis of COVID-19 was based on the clinical symptoms, polymerase chain reaction (PCR) test for SARS-CoV-2 from the nasopharyngeal swab, and/or radiological findings. We also considered the patients whose first swab PCR test was negative, but the repeated test was positive, to be confirmed cases. Moreover, the patients whose clinical and radiological findings were consistent with COVID-19, but swab PCR tests were negative or not available, were also considered as “probable COVID-19 patients” and were included in this study [13]. The diagnosis was made with swab PCR positivity in 72 patients (66.1%). In 37 patients (33.9%), swab PCR was negative. Laboratory tests (such as CRP, LDH, AST, and complete blood counts) of the patients were monitored daily during their hospitalization period (including ICU stay). It was analyzed to obtain target levels.

**Fig. 1:**
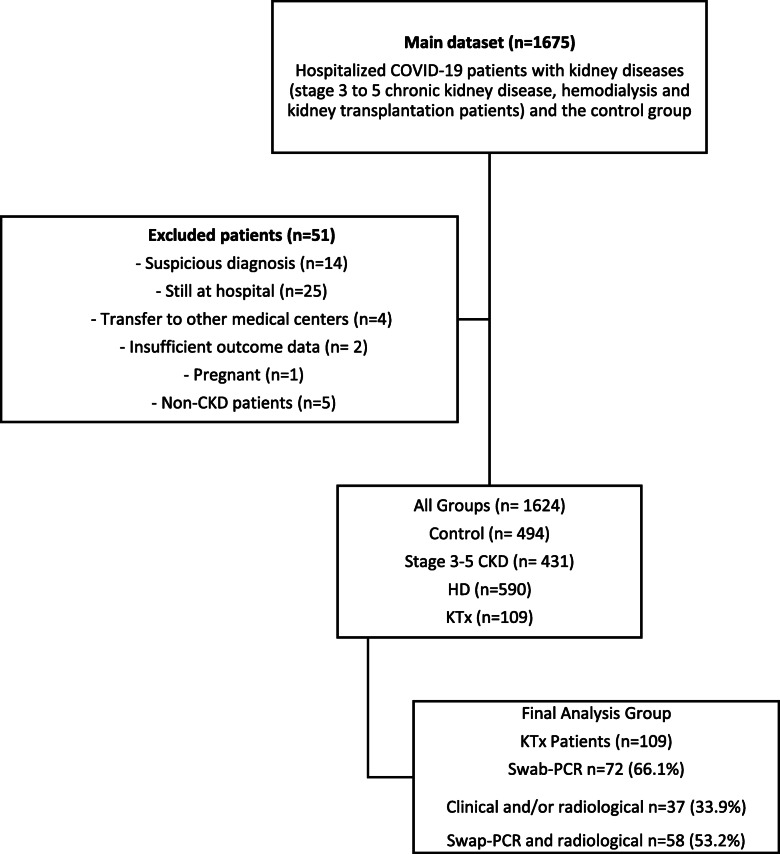
Flow chart illustrating the selection of the study population. Abbreviations: CKD: chronic kidney disease, HD: hemodialysis, KTx: kidney transplantation, PCR: polymerase chain reaction

We excluded the patients who were pregnant, younger than 18 years of age, lack hospital discharge or survival data, were still hospitalized at the time of data collection, and the patients hospitalized for non-COVID-19 reasons from this study. The study was approved by the University of Health Sciences, Istanbul Haseki Training and Research Hospital Ethics Committee (number 41–2020). The selection of the study population is shown in Fig. 1.

### Data collection

All participating centers scanned the electronic health records in hospital systems and recorded the data. We collected the admission data, including demographic information, duration of symptoms from onset to hospital admission, smoking habits, comorbidities and medications, initial serum creatinine, serum albumin, ferritin, C-reactive protein (CRP), hemoglobin, lymphocyte, and platelet counts. Dataset also included the clinical severity of COVID-19, laboratory tests performed during hospitalization, treatment for COVID-19 at the hospital, and the outcomes. COVID-19 severity was classified according to the suggestions in our national guideline [14]. The clinical severity of COVID-19 was defined according to the clinical presentation of COVID-19 at hospital admission and separated into four categories: patients with mild clinical symptoms without dyspnea or any sign of viral pneumonia on chest computerized tomography (CT) findings were defined as the mild disease, and patients with symptoms like fever and cough, dyspnea and signs of viral pneumonia on chest CT findings were as the moderate disease. The term severe disease referred to the patients requiring oxygen support on admission and the term critical disease referred to the patients who were hypoxic at admission and requiring close monitoring and/or need intensive care unit (ICU).

In terms of changes in immunosuppression, firstly, antimetabolite agents were discontinued. Calcineurin inhibitors (CNI) treatments were discontinued or the doses were reduced according to the severity of the disease in KTx patients (Tables 1, 2 and 3). No changes were made in immunosuppressive treatments in patients that were considered as “mild case” at admission (n: 3, 3.8%). However, MPA/AZA was discontinued in mild cases whose clinical condition did not improve within 3–5 days or in patients considered to be “moderate case” at the time of admission (n: 92, 84.4%). In patients with severe/critical COVID-19, all immunosuppressive drugs except steroids were discontinued (n: 14, 12.8%). All modifications were made by the investigator’s initiative.

**Table 1:** Baseline characteristics, lab tests, medication, and follow-up parameters of the patients according to survival

Characteristic		All patients *N* = 109	Survivors (*N* = 95)	Non-Survivors (*N* = 14)	*p*-value
Gender, Male, n (%)		63 (57.8)	57 (60.0)	6 (42.9)	0.225
Age (years), mean ± SD		48.4 ± 12.4	47.9 ± 12.4	51.7 ± 12.4	0.334
Donor type, n, (%)	Deceased	17 (15.6)	14 (14.7)	3 (21.4)	0.519
	Living	92 (84.4)	81 (85.3)	11 (78.6)	
Age > 60 years, n (%)		21 (19.4)	16 (76.2)	5 (23.8)	0.065
Time from symptom onset to admission, days, median (IQR)		4.5 (3.0–7.0)	5.0 (3.0–7.0)	4.0 (3.0–8.5)	0.634
LOS (days), median (IQR)		9.0 (6.0–14.0)	8.0 (6.0–13.5)	14 (8.0–17.5)	0.038
Time to from symptom onset to death or discharge, days, median (IQR)		14.0 (10.0–21.0)	13.0 (10.0–20.0)	21.0 (13.0–25.0)	0.092
Tx Duration, years, median (IQR)		5.0 (3.0–9.0)	5.0 (2.9–9.0)	5 (3.0–8.0)	0.899
Tx Duration < 1 year n (%)		17 (15.6)	15 (15.8)	2 (14.3)	0.885
Coexisting disorder, n/N (%)	Diabetes mellitus	25/107 (23.4)	21/93 (22.6)	4/14 (28.6)	0.621
	Hypertension	81/106 (76.4)	70/92 (76.1)	11/14 (78.6)	0.838
	Ischemic heart disease	18/103 (17.5)	13/90 (15.3)	5/13 (38.5)	0.033
	Heart failure	4/105 (3.8)	2/91 (2.2)	2/14 (14.3)	0.033
	COPD	5/105 (4.8)	4/92 (4.6)	1/13 (7.7)	0.491
	Cancer	6/105 (5.7)	6/92 (6.9)	0/13 (0)	1.000
	Chronic liver disease	1/105 (1.0)	1/92 (1.1)	0 (0)	1.000
Cause of kidney disease, n (%)	Diabetic nephropathy	13 (11.9)	10 (10.5)	3 (21.4)	0.764
	Glomerular disease	13 (11.9)	9 (9.4)	4 (28.6)	
	Hypertensive nephrosclerosis	28 (25.7)	25 (26.3)	3 (21.4)	
	ADPCKD	5 (4.6)	5 (5.2)	0 (0)	
	Amyloidosis	4 (3.7)	4 (4.2)	0 (0)	
	Chronic pyelonephritis	6 (5.5)	6 (6.3)	0 (0)	
	Urological diseases	6 (5.5)	6 (6.3)	0 (0)	
	Unknown	31 (28.4)	27 (28.4)	4 (28.5)	
	Others	3 (2.7)	3 (3.1)	0 (0)	
Smoking, n (%)	Former smoked	22 (20.2)	20/90 (22.2)	2/14 (14.3)	0.834
	Never smoker	43 (39.4)	36/90 (40.0)	7/14 (50.0)	
	Current smoker	1 (0.9)	1/90 (1.1)	0/14 (0)	
	Unknown	43 (39.4)	38/90 (42.2)	5/14 (35.7)	
Medications, n/N (%)	ACEi	21/103 (20.4)	17/85 (20.0)	4/14 (28.6)	0.414
	ARBs	14/102(13.7)	14/87(16.1)	0/14(0)	0.108
	Statins	11/101 (10.9)	9 (9.5)	2 (14.0)	0.308
	Anticoagulant or antiplatelet agent	45/102 (44.1)	37 (41.1)	8 (66.7)	0.094
	Oral antidiabetics	8/102 (7.8)	7 (8.0)	1 (7.4)	0.916
	Tacrolimus	86/109 (78.9)	75 (78.9)	11 (78.6)	0.974
	CsA	9/109 (8.3)	9 (9.5)	0 (0)	0.229
	MPA derivatives	94/109 (86.2)	80 (84.2)	14 (100)	0.109
	MTORi	12/109 (11.0)	10 (10.5)	2 (14.3)	0.651
	Azathioprine	6/103 (5.5)	6 (6.3)	0 (0)	0.333
	Prednisone	106/109 (97.2)	92 (96.8)	14 (100)	1.000
Induction therapy, yes, n, (%)		80 (73.4)	71 (74.7)	9 (64.3)	0.409
Induction therapy n, (%)	ATLG	67 (61.5)	61 (64.2)	6 (42.9)	0.270
	Basiliximab	13 (11.9)	10 (10.5)	3 (21.4)	
Modification of immunosuppression, n, (%)	No changed	3 (2.8)	3 (3.2)	0 (0.0)	0.163
	CNI withdrawal	0 (0.0)	0 (0.0)	0 (0.0)	
	MPA/AZA withdrawal	92 (84.4)	82 (86.3)	10 (71.4)	
	CNI + MPA/AZA withdrawal	14 (12.8)	10 (10.5)	4 (28.6)	
COVID-19 related clinic presentation at the time of diagnosis, n (%)	Mild disease	67 (61.4)	65 (59.6)	2 (14.3)	< 0.001
	Moderate Disease	33 (30.3)	27 (28.4)	6 (42.9)	
	Severe-Critical Disease	9 (8.3)	3 (3.2)	6 (42.9)	
Presentation symptoms n, (%)	Fever	70 (64.2)	60 (63.2)	10 (71.4)	0.547
	Myalgia	32 (29.4)	29 (30.5)	3 (21.4)	0.485
	Dyspnea	53 (48.6)	41 (43.2)	12 (85.7)	0.003
	Diarrhea	12 (11.0)	11 (11.6)	1 (7.1)	0.621
	Cough	72 (66.1)	64 (67.4)	8 (57.1)	0.451
	Throat pain	6 (5.5)	5 (5.3)	1 (7.1)	0.773
	Headache	14 (12.8)	14 (14.7)	0 (0.0)	0.124
	Fatigue	47 (43.1)	38 (40.0)	9 (64.3)	0.504
COVID-19 drug treatments, n/N (%)	Macrolides	71/106 (67.0)	60 (64.5)	11 (84.6)	0.149
	Oseltamivir	59/105 (56.2)	50 (54.3)	9 (69.2)	0.311
	Hydroxychloroquine	108/109 (99.1)	94 (98.9)	14 (100.0)	0.700
	Lopinavir-ritonavir	10/94 (10.6)	10 (12.2)	0 (.0)	0.201
	Favipiravir	49/100 (49.0)	38 (43.2)	11 (91.7)	0.002
	Glucocorticoids	59/101 (58.4)	47 (54.0)	12 (85.7)	0.026
	Tocilizumab	10 /99 (10.1)	5 (5.7)	5 (41.7)	< 0.001
	Anakinra	3/100 (3.0)	3(3.4)	0(.0)	0.497
	Apheresis / immunoadsorption	3/100 (3.0)	2 (2.3)	1(7.7)	0.357
Laboratory findings at admission, median (IQR)	Creatinine (μmol/l)	132.6 (97.2–194.5)	132.6 (97.2–181.2)	198.0 (97.2–293.7)	0.018
	Albumin (g/dl)	3.8 (3.4–4.1)	3.8 (3.4–4.0)	3.5 (3.0–3.83)	0.170
	Ferritin (μg/l)	369.5 (152–906)	338.0 (132–891)	679 (265–1718)	0.112
	Hemoglobin (g/dl) mean ± SD	11.6 ± 2.3	11.5 ± 2.4	12.0 ± 1.9	0.195
	Lymphocyte count (/μl)	850 (541–1257)	850 (540–1330)	790(557–1015)	0.412
	Lymphopenia (< 800 /μL) n, (%)	53 (48.6)	46 (48.4)	7 (50.0)	0.912
	Platelet count (×10^3^/μl)	199 (171–245)	199 (170–248)	189 (158–239)	0.474
Follow-up parameters	ICU admission, n (%)	22 (20.2)	9 (40.9)	13 (59.1)	< 0.001
	Bacterial superinfection, n, (%)	9 (8.3)	7 (7.4)	2 (14.3)	0.416
	Mechanical ventilation in ICU, n (%)	19 (17.4)	6 (31.6)	13 (68.4)	0.025
	Acute kidney injury, n (%)	46 (42.2)	36 (37.9)	10 (71.4)	0.018
	RRT, n (%)	4 (3.7)	1 (1.1)	3 (21.4)	< 0.001
	Leukopenia (< 4.0 × 10^3^/μl)	36 (33.0)	33(34.7)	3(21.4)	0.323
	Lymphopenia (< 800 /μl)	77/108 (71.3)	66 (69.5)	11(84.6)	0.258
	Thrombocytopenia (< 150 × 10^3^/μL)	16 (14.7)	13 (13.7)	3(21.4)	0.445
	LDH (> 2 × upper limit of normal)	29/104 (27.9)	21 (23.1)	8 (61.5)	0.004
	AST (> 2 × upper limit of normal)	15/98 (15.3)	10 (11.8)	5 (38.5)	0.013
	CRP (> 10 × upper limit of normal)	47 (43.1)	37 (38.9)	10 (71.4)	0.022

*p*-values presented from the chi-square test, Fisher’s exact test, t-test, or Mann-Whitney U test

*Abbreviations: IQR* interquartile range, *LOS* length of stay in the hospital, *COPD* chronic obstructive pulmonary disease, *ADPCKD* autosomal dominant polycystic kidney disease, *CsA* cyclosporine A, *ACEi* angiotensin-converting enzyme inhibitors, *ARBs* angiotensin II receptor blocker, *mTORi* mammalian target of rapamycin inhibitors, *MPA* mycophenolate derivatives, *CNI* calcineurin inhibitors, *AZA* azathioprine, *ATLG* anti-T lymphocyte globulin, *RRT* renal replacement therapy, *CRP* C reactive protein, *LDH* lactate dehydrogenase, *AST* aspartate aminotransferase, *ICU* intensive care unit

**Table 2:** Baseline characteristics, lab tests, medication, and follow-up parameters of the patients according to ICU admission

		ICU admission		*p*-value
Characteristic		No *N* = 87	Yes *N* = 22	
Demographic information				
Male Gender, n (%)		52 (59.8)	11 (50.0)	0.407
Age (years), median (IQR)		48 (38.0–56.0)	51 (44.0–64.0)	0.227
Donor type, n, (%)	Deceased	11 (12.6)	6 (27.3)	0.091
	Living	76 (87.4)	16 (72.7)	
> 60 years n, %		13 (14.9)	8 (38.1)	0.016
Time from symptom onset to admission, days, median (IQR)		4 (3.0–7.0)	5 (3.0–7.0)	0.536
Transplantation duration, years, median (IQR)		5 (3.0–9.0)	6 (3.0–9.0)	0.774
Length of stay in hospital (days), median (IQR)		9 (6.0–13.0)	14.5 (8.0–18.0)	0.003
Tx Duration < 1 year n (%)		14 (16.1)	3 (13.6)	0.777
Coexisting disorder, n/N (%)	Diabetes mellitus	18/85 (21.2)	7/22 (31.8)	0.293
	Hypertension	62/84 (73.8)	19/22 (86.4)	0.217
	Ischemic heart disease	12/82 (14.6)	6/21 (28.6)	0.133
	Heart failure	1/83 (1.2)	3/22 (13.6)	0.007
	COPD	3/84 (3.6)	2/21 (9.5)	0.252
	Cancer	6/84 (7.1)	0/21 (0.0)	0.207
	Chronic liver disease	4/84 (4.6)	1/21 (4.5)	0.615
Cause of kidney disease, n (%)	Diabetic nephropathy	4 (4.6)	0 (0.0)	0.189
	Glomerular disease	6 (6.9)	0 (0.0)	
	Hypertensive nephrosclerosis	9 (10.3)	4 (18.2)	
	ADPCKD	8 (9.2)	5 (22.7)	
	Amyloidosis	22 (25.3)	6 (27.3)	
	Chronic pyelonephritis	0 (0.0)	1 (4.5)	
	Urological diseases	2 (2.3)	0 (0.0)	
	Unknown	26 (29.9)	5 (22.7)	
	Others	6 (6.9)	0 (0.0)	
Smoking, n (%)	Former smoked	18 (20.7)	4 (18.2)	0.890
	Never smoker	33 (37.9)	10 (45.5)	
	Current smoker	1 (1.1)	0 (0.0)	
	Unknown	35 (40.2)	8 (36.4)	
Medications, n/N (%)	ACEi	17/82 (20.7)	4/21 (19.0)	0.864
	ARBs	12/81 (14.8)	2/21 (9.5)	0.530
	Statins	7/82 (8.5)	4/19 (21.1)	0.115
	Anticoagulant or antiplatelet agent	34/83 (41.0)	11/19 (57.9)	0.180
	Oral antidiabetics	7/81 (8.6)	1/21 (4.8)	0.556
	Tacrolimus	70 (80.5)	16 (72.7)	0.427
	CsA	9 (10.3)	0 (0.0)	0.115
	MPA derivatives	75 (86.2)	19 (86.4)	0.985
	MTORi	9 (10.3)	3 (13.6)	0.659
	Azathioprine	5 (5.7)	1 (4.5)	0.825
	Prednisone	85 (97.7)	21 (95.5)	0.565
Induction, yes, n, (%)		67 (77.0)	13 (52.1)	0.089
Induction therapy n, (%)	ATLG	57 (65.5)	10 (45.5)	0.189
	Basiliximab	10 (11.5)	3 (13.6)	
Modification of immunosuppression n, (%)	No changed	3 (2.8)	0 (0.0)	0.059
	MPA/AZA withdrawal	76 (87.4)	16 (72.7)	
	CNI + MPA/AZA withdrawal	8 (9.2)	6 (27.3)	
COVID-19 related clinic presentation at the time of diagnosis, n (%)	Mild disease	64 (73.5)	3 (13.6)	< 0.001
	Moderate Disease	22 (23.5)	11 (50.0)	
	Severe-Critical Disease	1 (1.1)	8 (36.4)	
Presentation symptoms n, (%)	Fever	55 (63.2)	15 (68.2)	0.664
	Myalgia	27 (31.0)	5 (22.7)	0.445
	Dyspnea	36 (41.4)	17 (77.3)	0.003
	Diarrhea	11 (12.6)	1 (4.5)	0.278
	Cough	60 (69.0)	12 (54.5)	0.202
	Throat pain	4 (4.6)	2 (9.1)	0.409
	Headache	11 (12.6)	3 (13.6)	0.901
	Fatigue	36 (41.4)	11 (50.0)	0.466
COVID-19 drug treatments, n/N (%)	Macrolides	55 (64.7)	16 (76.2)	0.316
	Oseltamivir	45 (53.6)	14 (66.7)	0.279
	Hydroxychloroquine	86 (98.9)	22 (100.0)	0.613
	Lopinavir-ritonavir	8 (10.4)	2 (11.8)	0.868
	Favipiravir	31 (38.3)	18 (94.7)	< 0.001
	Glucocorticoids	41 (51.3)	18 (85.7)	0.004
	Tocilizumab	5 (6.3)	5 (26.3)	0.009
	Anakinra	3 (3.8)	0 (0.0)	0.379
	Apheresis / immunoadsorption	0 (0.0)	3 (15.0)	< 0.001
Laboratory findings at admission, median (IQR)	Creatinine (μmol/l)	132.6 (88.4–176.8)	198.0 (106.1–265.2)	0.016
	Albumin (g/dl)	3.8 (3.5–4.1)	3.45 (2.9–3.8)	0.003
	Ferritin (μg/l)	328 (129.0–814.0)	728 (514.0–2000.0)	0.029
	Hemoglobin (g/dl) mean ± SD	11.6 (10.0–13.3)	11.4 (9.7–13.5)	0.970
	Lymphocyte count (/μl)	860 (547.0–1380.0)	705 (460.0–950.0)	0.086
	Lymphopenia (< 800 /μl) n. (%)	41 (47.1)	12 (54.5)	0.534
	Platelet count (×10^3^/μl)	200 (170.0–249.0)	185 (161.0–232.0)	0.275
Follow-up parameters, n (%)	Acute kidney injury, n (%)	31 (35.6)	15 (68.2)	0.006
	RRT, n (%)	0 (0.0)	4 (18.2)	< 0.001
	Bacterial superinfection. N (%)	6 (6.9)	3 (13.6)	0.527
	Laboratory tests during hospitalization, n (%)			
	Leucopenia (< 4.0 /μl)	28 (32.2)	8 (36.4)	0.710
	Lymphopenia (800 /μl)	57 (66.3)	20 (90.9)	0.023
	Thrombocytopenia (< 150 × 10^3^/ μl)	11 (12.6)	5 (22.7)	0.232
	LDH (> 2 × upper limit of normal)	13 (15.7)	16 (76.2)	< 0.001
	AST (> 2 × upper limit of normal)	7 (9.1)	8 (38.1)	0.001
	CRP (> 10 × upper limit of normal)	31 (35.6)	16 (72.7)	0.002
The final situation, n (%)	Recover	86 (98.9)	9 (40.9	< 0.001
	Exitus	1 (1.1)	13 (59.1)	

*p*-values presented from the chi-square test, Fisher’s exact test, t-test, or Mann-Whitney U test

*Abbreviations: IQR* interquartile range, *LOS* length of stay in the hospital, *COPD* chronic obstructive pulmonary disease, *ADPCKD* autosomal dominant polycystic kidney disease, *CsA* cyclosporine A, *ACEi* angiotensin-converting enzyme inhibitors, *ARBs* angiotensin II receptor blocker, *mTORi* mammalian target of rapamycin inhibitors, *MPA* mycophenolate derivatives, *CNI* calcineurin inhibitors, *AZA* azathioprine, *ATLG* anti-T lymphocyte globulin, *RRT* renal replacement therapy, *CRP* C reactive protein, *LDH* lactate dehydrogenase, *AST* aspartate aminotransferase, *ICU* intensive care unit

**Table 3:** Baseline characteristics, lab tests, medication, and follow-up parameters of the patients according to the secondary outcome (dead and/or ICU admission)

		Secondary outcome (dead and/or ICU admission)		*p*-value
Characteristic		No *N* = 86	Yes *N* = 23	
Demographic information				
Male Gender, n (%)		52 (60.5)	12 (52.2)	0.276
Age (years), median (IQR)		48 (38–56)	55 (44–64)	0.085
Donor type, n, (%)	Deceased	11 (12.8)	6 (26.1)	0.118
	Living	75 (87.2)	17 (73.9)	
> 60 years n, %		12 (13.9)	9 (39.1)	0.004
Time from symptom onset to admission, days, median (IQR)		4.0 (3.0–7.0)	5.0 (3.0–7.0)	< 0.001
Transplantation duration, years, median (IQR)		5.0 (3.5–9.25)	6.0 (3.0–9.5)	0.545
Length of stay in hospital (days), median (IQR)		8.5(6.0–13.0)	14.0 (8.0–18.5)	< 0.001
Tx Duration < 1 year n (%)		13 (15.1)	4 (17.4)	0.789
Coexisting disorder, n/N (%)	Diabetes mellitus	18/84 (21.4)	7/23 (30.4)	0.366
	Hypertension	61 (73.5)	20 (87.0)	0.178
	Ischemic heart disease	11/81 (13.6)	7/22 (31.8)	0.046
	Heart failure	1/81 (1.2)	3/23 (13.0)	0.009
	COPD	3/83 (3.6)	2/22 (9.1)	0.284
	Cancer	6/83 (7.2)	0(0.0)	0.194
	Chronic liver disease	1/83 (1.2)	0 (0.0)	0.605
Cause of kidney disease, n (%)	Diabetic nephropathy	9 (10.5)	4 (17.4)	0.231
	Glomerular disease	8 (9.3)	5 (21.7)	
	Hypertensive nephrosclerosis	22 (25.6)	6 (26.1)	
	ADPCKD	4 (4.7)	1 (4.3)	
	Amyloidosis	4 (4.7)	0 (0.0)	
	Chronic pyelonephritis	6(7.0)	0 (0.0)	
	Urological diseases	6 (7.0)	0 (0.0)	
	Unknown	25 (29.1)	6 (26.1)	
	Others	2 (2.3)	0 (0.0)	
Smoking, n (%)	Former smoked	18 (20.9)	4 (17.4)	0.919
	Never smoker	34 (39.5)	9 (39.1)	
	Current smoker	1 (1.2)	0 (0.0)	
	Unknown	33 (38.4)	10 (43.5)	
Medications, n/N (%)	ACEi	17/81 (21.0)	4/22 (18.2)	0.772
	ARBs	12/80 (15.0)	2/22 (9.1)	0.476
	Statins	7/81 (8.6)	4/20 (20.0)	0.144
	Anticoagulant or antiplatelet agent	33/82 (40.2)	12/20 (60.0)	0.111
	Oral antidiabetics	7/80 (8.8)	1/22 (4.5)	0.516
	Tacrolimus	69 (80.2)	17 (73.9)	0.509
	CsA	9 (10.5)	0 (0.0)	0.105
	MPA derivatives	12 (14.0)	20 (87.0)	0.910
	MTORi	9 (10.5)	3 (13.0)	0.726
	Azathioprine	5/81 (5.8)	1/22 (4.3)	0.784
	Prednisone	84 (97.7)	22 (95.7)	0.599
Induction therapy, yes, n, (%)		66 (76.7)	14 (60.9)	0.126
Induction therapy n, (%)	ATLG	56 (65.1)	11 (47.8)	0.268
	Basiliximab	10 (11.6)	3 (13.0)	
Modification of immunosuppression n, (%)	No changed	3 (3.5)	0 (0.0)	0.076
	MPA/AZA withdrawal	75 (87.2)	17 (73.9)	
	CNI + MPA/AZA withdrawal	8 (9.3)	6 (26.1)	
COVID-19 related clinic presentation at the time of diagnosis, n (%)	Mild disease	64 (74.4)	3 (13.0)	< 0.001
	Moderate Disease	22 (25.6)	11 (47.8)	
	Severe-Critical Disease	0 (0.0)	9 (39.1)	
Presentation symptoms n, (%)	Fever	54 (62.8)	16 (69.6)	0.547
	Myalgia	27 (31.4)	5 (21.7)	0.366
	Dyspnea	35 (40.7)	18 (78.3)	0.001
	Diarrhea	11 (12.8)	1 (4.3)	0.251
	Cough	59 (68.6)	13 (56.5)	0.277
	Throat pain	4 (4.7)	2 (8.7)	0.450
	Headache	11 (12.8)	3 (13.0)	0.974
	Fatigue	35 (40.7)	10 (43.5)	0.324
COVID-19 drug treatments, n/N (%)	Macrolides	54/84 (64.3)	17/22 (77.3)	0.249
	Oseltamivir	44 (53.0)	15/22 (68.2)	0.202
	Hydroxychloroquine	85 (98.8)	23 (100.0)	0.603
	Lopinavir-ritonavir	8/76 (10.5)	2/18 (11.1)	0.942
	Favipiravir	30/80 (37.5)	19/20 (95.0)	< 0.001
	Glucocorticoids	40/79 (50.6)	19/22 (86.4)	0.003
	Tocilizumab	4/79 (5.1)	6/20 (30.0)	0.001
	Anakinra	3/76 (3.8)	0 (0.0)	0.365
	Apheresis / immunoadsorption	0 (0.0)	3/21 (14.3)	0.001
Laboratory findings at admission, median (IQR)	Creatinine (μmol/l)	132.6 (89.7–177.7)	441.3 (262.5–735.5)	0.050
	Albumin (g/dl)	3.8 (3.5–4.1)	3.4 (2.9–3.8)	0.002
	Ferritin (μg/l)	331(128–839)	679 (184–2260)	0.048
	Hemoglobin (g/dl) mean ± SD	11.6 ± 2.4	11.6 ± 2.1	0.900
	Lymphocyte count (/μl)	860 (545–1385)	700 (460–950)	0.018
	Lymphopenia (< 800 /μl) n, (%)	57 (66.3)	20 (90.9)	0.394
	Platelet count (×10^3^/μl)	199 (169–248)	186 (161–239)	0.451
Follow-up parameters, n (%)	Acute kidney injury, n (%)	31 (36.0)	15 (65.2)	0.012
	RRT, n (%)	0 (0.0)	4 (17.4)	< 0.001
	Bacterial superinfection, n (%)	6 (7.0)	3 (13.0)	0.348
	Laboratory tests during hospitalization, n (%)			
	Leucopenia (< 4.0 /μl)	28 (32.6)	8 (34.8)	0.840
	Lymphopenia (800 /μl)	57(66.3)	20 (90.9)	0.023
	Thrombocytopenia (< 150 × 10^3^/ μl)	11 (12.8)	5 (21.7)	0.281
	LDH (> 2 × upper limit of normal)	13 (15.9)	16 (72.7)	< 0.001
	AST (> 2 × upper limit of normal)	7 (9.2)	8 (36.4)	0.002
	CRP (> 10 × upper limit of normal)	30 (34.9)	17 (73.9)	0.001
The final situation, n (%)	Recover	86 (100.0)	9 (22.2)	< 0.001
	Exitus	0 (0)	14 (77.8)	

*p*-values presented from the chi-square test, Fisher’s exact test, t-test, or Mann-Whitney U test

*Abbreviations: IQR* interquartile range, *LOS* length of stay in the hospital, *COPD* chronic obstructive pulmonary disease, *ADPCKD* autosomal dominant polycystic kidney disease, *CsA* cyclosporine A, *ACEi* angiotensin-converting enzyme inhibitors, *ARBs* angiotensin II receptor blocker, *mTORi* mammalian target of rapamycin inhibitors, *MPA* mycophenolate derivatives, *CNI* calcineurin inhibitors, *AZA* azathioprine, *ATLG* anti-T lymphocyte globulin, *RRT* renal replacement therapy, *CRP* C reactive protein, *LDH* lactate dehydrogenase, *AST* aspartate aminotransferase, *ICU* intensive care unit

### Outcomes

The primary outcomes were in-hospital mortality and the need for ICU admission. The secondary outcome was composite in-hospital mortality and/or ICU admission. Length of stay (LOS) at the hospital was used in the in-hospital mortality analyses, which was defined as the period starting from the day of hospitalization and ending on the day of death, admission to the ICU, or discharge. Acute kidney injury (AKI) was defined by the following criteria determined by KDIGO guidelines: increase in serum creatinine ≥0.3 mg/dl or increase in serum creatinine to > 1.5 times the baseline creatinine levels [15]. The need for renal replacement therapy (RRT) and the requirement of invasive mechanical ventilation (IMV) for patients admitted in the ICU were recorded.

### Statistical analysis

The analyses were performed using the IBM SPSS Statistics for Windows, Version 23.0 (IBM Corp., Armonk, NY, USA). We summarized descriptive statistics as numbers and percentages for categorical variables, and mean, standard deviation, median, minimum-maximum, and interquartile range (IQR) for numerical variables, where appropriate. For the comparisons of categorical variables, the chi-square test or Fisher’s exact test (when expected frequencies for some cells are < 5) were used. We used Student’s t-test to compare the two independent groups in the analyses of the normally distributed numerical data, and the Mann-Whitney-U test in the case of abnormal distribution of numerical data. To find out the independent parameters related to primary and secondary outcomes, we created a logistic regression analysis model with the entering method using parameters that included demographic, clinical, and laboratory parameters that suggested a potential effect on the outcomes in univariate analyses. Parameters with *p* < 0.05 in univariate analyzes were considered significant and they were included in multivariate analyzes. A *P*-value of < 0.05 was considered significant.

## Results

### Demographic and clinical characteristics

A total of 109 KTx recipients hospitalized with COVID-19 from 34 different centers were included in the study. 63 (57.8%) were male, and the mean age was 48.4 ± 12.4 (19.4% more than 60) years (Table 1). Hypertension was the most common coexisting disorder affecting 76.4% of patients, followed by diabetes mellitus (23.4%), ischemic heart disease (17.5%), cancer (5.7%), and chronic obstructive pulmonary disease (COPD) (4.8%). 21.1% of the patients had a previous or current smoking history. The median time between transplantation and the diagnosis of COVID-19 was 5.0 (IQR 3.0–9.0) years. Table 1 shows the baseline characteristics of patients according to survival.

### Clinical outcome

Median LOS was 9 days (IQR: 6–14 days). AKI developed in 46 (42.2%), and 4 (3.7%) patients needed RRT. A total of 22 (20.2%) patients were admitted to ICU, and 19 (17.4%) patients required IMV. The development of AKI (71.4% vs. 37.9% respectively; *p* = 0.018), requiring IMV (68.4% vs. 31.6% respectively; *p* = 0.025), and need for RRT (21.4% vs. 1.1%, respectively; *p* < 0.001) were significantly higher in non-survivors compared to survivors. A total of 14 (12.8%) patients died. 23 (21.1%) patients reached the secondary outcome.

Parameters found to be associated with primary outcome and secondary outcome.

Ischemic heart disease and heart failure were higher in patients who died than surviving patients (38.5% vs. 15.3%, *p* = 0.033; 14.3% vs. 2.2%, *p* = 0.028, respectively) and those reaching secondary outcome (31.8% vs 13.6%, *p* = 0.046; 13.0% vs 1.2%, *p* = 0.009, respectively) (Tables 1,3). Non-survivor patients had longer LOS than other patients [14 days (IQR: 8–17.5 days) vs. 8 days (IQR: 6–13.5 days), *p* = 0.038] (Table 1). ICU needs were observed significantly more frequently in patients with heart failure compared to other patients (Table 2).

Neither age, gender, transplantation duration, primary kidney disease, comorbidities (except as mentioned above), smoking history, maintenance immunosuppression nor use of angiotensin-converting enzyme inhibitors or angiotensin receptor blockers (ACEi/ARBs) was significantly different between the patients reaching primary and secondary outcomes (Tables 1,2,3).

Patients reaching the secondary outcome had longer LOS than other patients (14 days [IQR: 8–18.5 days] vs. 8.5 days [IQR: 6–13 days], *p* < 0.001) (Table 3).

### Presentation, laboratory results, and treatment according to primary and secondary outcomes

The most common symptoms at admission were coughing (66.1%) and fever (64.2%), followed by.

dyspnea (48.6%) and fatigue (43.1%). The presence of dyspnea (85.7% vs. 43.2%, *p* = 0.003) at admission was significantly higher in non-survivors compared to survivors. Most of the patients (60.6%) had a mild disease at the time of admission (Table 1).

48.6% of the patients had lymphopenia (< 800 /μl) at admission, but neither the lymphopenia nor the lymphocyte count was significantly different between the survivor and non-survivor patients. Serum creatinine [198.0 μmol/l (IQR: 97.2–293.7 μmol/l) vs. 132.6 μmol/l (IQR: 97.2–181.2 μmol/l) respectively, *P* = 0.018], CRP levels (during follow-up period, > × 10 upper limit) were significantly higher (71.4% vs. 38.9%, *p* = 0.022) in non-survivors than survivors. Non-survivor patients had significantly higher (more than 2 times increase in the upper limit of normal) serum lactate dehydrogenase (LDH) (61.5% vs. 23.1%, *p* = 0.004) and aspartate transaminase (AST) levels (38.5% vs. 11.8%, *p* = 0.013).

There was no statistically significant difference between the two groups in ferritin, hemoglobin, platelet, and serum albumin levels (Table 1).

Serum creatinine [198.0 μmol/l (IQR: 106.1–265.2 μmol/l) vs 132.6 μmol/l (IQR: 88.4–176.8 μmol/l) vs. respectively, *P* = 0.016], ferritin level [728 μg/l (IQR 514.0–2000.0 μg/l) vs 328 μg/l (IQR 129.0–814.0 μg/l) respectively, *p* = 0.029), CRP levels (during follow-up period, > × 10 upper limit) (72.7% vs. 35.6%, respectively, *p* = 0.002), LDH levels (during follow-up period, > × 2 upper limit) (76.2% vs 15.7%, respectively, *p* < 0.001), AST levels (during follow-up period, > × 2 upper limit) (38.1% vs 9.1%, respectively, *p* = 0.001) were significantly higher in patients followed in the ICU than others. However, serum albumin levels were significantly lower [3.45 g/dl (IQR 2.9–3.8 g/dl) vs 3.8 g/dl (IQR 3.5–4.1 g/dl), respectively, *p* = 0.003] in patients followed in the ICU compared to the others. There was no statistically significant difference between the two groups in terms of hemoglobin, thrombocyte, and lymphocyte count at the time of admission (Table 2).

In terms of secondary outcome, serum creatinine levels [441.3 μmol/l (IQR: 262.5–735.5 μmol/l) vs. 1.5 μmol/l (IQR: 89.7–177.7 μmol/l) respectively, *p* = 0.05] serum ferritin levels [679 μg/l (IQR:184–2260 mg/dl) vs. 132.6 μg/l (IQR: 128–839 μg/l) respectively, *p* = 0.048], CRP levels (during follow up period, >× 10 upper limit,73.9% vs. 34.9%, *p* = 0.001) were significantly higher in patients who reached secondary outcome. Serum albumin level [3.4 g/dl (2.9–3.8) vs. 3.8 g/dl (3.5–4.1), *p* = 0.002] and presence of lymphopenia rate (< 800 /μl) (90.9% vs. 66.3% *p* = 0.018) were significantly lower in these patients. Patients who reached the secondary outcome had significantly higher (more than 2 times increase in the upper limit of normal) serum LDH (72.7% vs. 15.9%, *p* < 0.001) and AST levels (36.4% vs. 9.2%, p < 0.001), and significantly higher (more than 10 times increase in the upper limit of normal) CRP (73.9% vs. 34.9%, p = 0.001). There was no statistically significant difference in terms of leucopenia, thrombocytopenia during hospitalization period between groups (Table 3).

### Treatment of COVID-19

Almost all patients received hydroxychloroquine (99.1%), majority of the patients received macrolide (67%), oseltamivir (56.2%) glucorticoids (58.4%) and favipiravir (49.0%) while a smaller subset of the patients received tocilizumab (10.1%) or anakinra (3%) and lopinavir/ritonavir (10.6%) (Table 1). There was significant difference in mortality among tocilizumab (41.7% vs. 5.7%, p < 0.001), glucocorticoids (85.7% vs. 54%, *p* = 0.026) and favipiravir (91.7% vs. 43.2%, *p* = 0.002) treatments of COVID-19.

### Predictors of primary and secondary outcomes

In univariate analyzes, it was determined that the presence of ischemic heart disease, initial serum creatinine levels were associated with mortality. Both parameters were found to be predictive of mortality in multivariate analysis (Table 4).

**Table 4:** Univariate and multivariate logistic regression analysis of the parameters related to mortality

	Univariate Analysis		Multivariate Analysis	
	Odds Ratio (95% CI)	*p*-value	Odds Ratio (95% CI)	*p*-value
Age > 60 years	2.743 (0.811–9.274)	0.104		
Male gender	0.500 (0.161–1.556)	0.231		
Presence of diabetes mellitus	1.371 (0.390–4.822)	0.622		
Presence of hypertension	1.152 (0.295–4.506)	0.838		
Presence of ischemic heart disease	3.702 (1.047–13.083)	0.042	**4.129 (1.104–15.442)**	**0.035**
Initial lymphocyte count	0.999 (0.998–1.000)	0.222		
Initial serum ferritin level	1.000 (1.000–1.001)	0.373		
Initial serum albumin level	0.492 (0.178–1.360)	0.172		
Initial serum creatinine level	1.520 (1.016–2.274)	0.042	**1.681 (1.083–2.608)**	**0.021**

*Abbreviations: CI* confidence interval

In univariate analyzes, it was determined that older age (> 60 years), initial serum creatinine, ferritin, albumin levels, lymphocyte count were associated with ICU admission. However, older age and initial serum creatinine were found to be predictive in multivariate analysis (Table 5).

**Table 5:** Univariate and multivariate logistic regression analysis of the parameters related to the ICU admission

	Univariate Analysis		Multivariate Analysis	
	Odds Ratio (95% CI)	*p*-value	Odds Ratio (95% CI)	*p*-value
Age > 60 years	3.503 (1.214–10.108)	0.020	**5.754 (1.331–24.882)**	**0.019**
Male gender	0.673 (0.263–1.722)	0.409		
Presence of diabetes mellitus	1.737 (0.616–4.900)	0.297		
Presence of hypertension	2.247 (0.606–8.339)	0.226		
Presence of ischemic heart disease	2.333 (0.756–7.205)	0.141		
Initial lymphocyte count	0.999 (0.998–1.000)0	0.028	**0.999 (0.997–1.000)**	**0.111**
Initial serum ferritin level	1.000 (1.000–1.001)	0.042	**1.000 (1.000–1.001)**	**0.100**
Initial serum albumin level	0.281(0.111–0.714)	0.008	**0.864 (0.249–2.996)**	**0.818**
Initial serum creatinine level	1.747 (1.142–2.674)	0.010	**1.757 (1.016–3.036)**	**0.044**

*Abbreviations: CI* confidence interval

In univariate analyzes, age over 60 years, baseline lymphocyte counts, initial serum creatinine and albumin levels were found to be predictive of secondary outcome. In multivariate analysis, age over 60 years and initial lymphocyte count were found to be related to secondary outcome (Table 6).

**Table 6:** Univariate and multivariate logistic regression analysis of the parameters related to secondary outcome (dead and/or ICU admission)

	Univariate Analysis		Multivariate Analysis	
	Odds Ratio (CI 95%)	*p*-value	Odds Ratio (CI 95%)	*p*-value
Age > 60 years	3.964 (1.407–11.171)	0.009	**4.123 (1.152–14.753)**	**0.029**
Male gender	1.668 (0.661–4.209)	0.278		
Presence of diabetes mellitus	1.604 (0.573–4.492)	0.368		
Presence of ischemic heart disease	2.404 (0.650–8.891)	0.189		
Presence of hypertension	2.404 (0.650–8.891	0.189		
Initial lymphocyte count	0.999 (0.998–1.000)	0.022	**0.999 (0.998–1.000)**	**0.046**
Initial serum ferritin level	1.000 (1.000–1.001)	0.062		
Initial serum albumin level	0.275 (0.109–0.694)	0.006	**0.638 (0.216–1.885)**	**0.416**
Initial serum creatinine level	1.630 (1.086–2.446)	0.018	**1.573 (0.989–2.502)**	**0.056**

*Abbreviations: CI* confidence interval

## Discussion

In our national registry, the mortality rate was 12.8% in KTx recipients hospitalized with COVID-19, unlike previous single-center reports that observed mortality rates of 24–30% [2, 16–18]. In multivariate analysis, age over 60 years, presence of ischemic heart disease, initial serum creatinine level and lymphocyte count were found to be predictors of disease severity and mortality.

COVID-19 mortality rates in the general population vary from center to center. In the first study of 191 patients from China, mortality rates were found to be 28% [5]. In subsequent publications, these rates were reported to be 8% in New York, 14% in Italy, and 12% in Spain [1]. According to the national data of our Ministry of Health (about 2.464.030 COVID-19 patients) hospitalized until 30.01.2021, the overall mortality rate is 2.49% [19]. It is unclear whether the mortality in the kidney or any SOT recipients is higher than in the general inpatient population. COVID-19 mortality in SOT recipients is higher than the normal population and also varies between 18 and 30% in different centers [2, 7, 16–18]. However, in a large cohort study evaluating patients hospitalized for COVID-19, mortality, need for ICU care, and mechanical ventilation support rates were similar between SOT recipients and non-transplant patients [20]. Another large study evaluating 482 SOT recipients with COVID-19 found that the overall mortality was similar to the general non-transplant patient population with similar comorbidities [21]. In a recent study evaluating KTx recipients with COVID-19, authors reported the AKI (52%), requiring IMV (29%) and overall mortality (32%) [22]. Compared to our patient group, AKI and IMV rates seem to be lower in our cohort. KTx recipients had a number of comorbid conditions such as hypertension, diabetes, ischemic heart disease. Although diabetes mellitus was associated with severe disease in both the TANGO study [22] group and the French cohort [23], the presence of hypertension alone was not associated with death in both studies. Ischemic heart disease is common in KTx recipients and is the leading cause of mortality [24]. In our series, the rates of diabetes mellitus, hypertension, and ischemic heart disease were lower compared to other studies. In this study, a significant relationship was found between ischemic heart disease and mortality, but the presence of hypertension and diabetes mellitus was not found to be associated with mortality. Although the French cohort found an association between the presence of cardiovascular disease and severe illness and death, no similar findings were reported in the TANGO study. Since our patients were younger compared to patients in other cohorts, the adverse effects of diabetes mellitus may have been reduced, and therefore the significant relationship between diabetes and mortality observed in other studies may not have been detected in this study. The overall low mortality in our series can be explained by the fact that our patients were younger than the other cohorts and the disease was less severe due to the low frequency of comorbidities. On comparing our KTx recipients with the normal population, mortality was found higher in KTx recipients (12.8% vs 2.49%) which is consistent with previous reports [2, 7, 16–18].

According to the treatment algorithm of the Ministry of Health, the use of favipiravir was limited only to intensive care patients during the period of the study. Therefore, this situation with favipiravir was attributed to selection bias.

Lymphopenia is common in the course of COVID-19 in both the general population and SOT recipients, and several studies have shown an association between disease severity and lymphopenia [20, 21, 25–27]. Our findings are in line with previous reports.

Cytokine storm is an important situation in the course of COVID-19 patients and is associated with death [28, 29]. Steroids and tocilizumab are used as treatments for this condition [30–32]. In the RECOVERY study, which is a randomized clinical study, it was determined that the addition of 6 mg dexamethasone to the usual treatment provided significant improvements in patients who needed oxygen or ventilator support [33]. Although some promising results have been reported in the early stages of the pandemic, later randomized trials revealed uncertainties regarding the efficacy of tocilizumab [31, 34]. In a recently published randomized placebo-controlled study in hospitalized patients with COVID-19 pneumonia, tocilizumab reduced the likelihood of mechanical ventilation or death to progress to the composite outcome, but no improvement in survival was found [35]. However, in a multicenter Spanish study evaluating 80 kidney transplant recipients with COVID-19, higher mortality was found in the group that received tocilizumab compared to those who did not [32]. We also did not observe any specific benefit from tocilizumab use.

However, it is difficult to interpret the negative results in patients receiving steroids and/or tocilizumab in our study. All patients were receiving steroids (methylprednisolone 60 mg/day) and tocilizumab concurrently, so it is not possible to determine whether the net effect were associated with these medications. Also, our patients who received tocilizumab had more severe illnesses and a higher rate of oxygen needs. Because of this patient selection bias regarding tocilizumab use, we could not reach on conclusion about the relationship between tocilizumab use and negative outcomes.

In COVID-19, the presence of smoking and COPD was associated with severe disease and mortality [36]. However, no increased risk associated with smoking or COPD was reported in either the TANGO study [22] or the French cohort [23]. Similarly, we did not find an association between COPD and smoking and adverse clinical outcomes.

Although its frequency varies between centers [37–39], AKI is common during the course of COVID-19 due to renal hypoperfusion, cytokine storm, and multi-organ failure. In our study, the frequency of AKI and RRT was 42.2 and 3.7%, respectively. Both AKI and RRT were associated with disease severity and mortality. The significant relationship between mortality and creatinine levels at admission show that graft functions have prognostic significance in KTx recipients with COVID-19. These results are consistent with the TANGO study [22] and the French cohort [23].

Although this multicenter study has a large sample size, it has many limitations mainly due to its retrospective nature. For this reason, associations of some parameters with mortality reported may not reflect the causal relationship. Changes in treatment algorithms during the patient recruitment phase made it difficult to evaluate the results. Problems related to patient selection made it difficult to evaluate the treatment results, such as in the tocilizumab use. We also included PCR negative patients as clinical diagnosis of COVID-19. This issue should be considered on evaluating results.

In conclusion, COVID-19 in KTx has a high mortality rate, especially in patients with ischemic heart disease and poor graft function. Low lymphocyte counts at admission and age over 60 years increased the risk for their combined endpoint of death or ICU admission.

## Data Availability

The datasets used and/or analyzed during the current study available from the corresponding author on reasonable request.

## Abbreviations

KTx: Kidney transplantation
COVID-19: Coronavirus-19 disease
SOT: Solid organ transplantation
PCR: Polymerase chain reaction
IMV: Invasive mechanical ventilation
CT: computarized tomography
CNI: Calcineurin inhibitors
LOS: Length of stay
IQR: Interquartile range
LOS: Length of stay in hospital
COPD: Chronic obstructive pulmonary disease
ADPCKD: Autosomal dominant polycystic kidney disease
CsA: Cyclosporine A
ACEi: Angiotensin-converting enzyme inhibitors
ARBs: Angiotensin II receptor blocker
mTORi: Mammalian target of rapamycin inhibitors
MPA: Mycophenolate derivates
RRT: Renal replacement therapy
CRP: C reactive protein
LDH: Lactate dehydrogenase
AST: Aspartate aminotransferase
ICU: Intensive care unit
AKI: Acute kidney injury
CI: Confidence intervals
